# Beyond the Algorithm: Artificial Intelligence, Clinical Decision-Making, and the Moral Ecology of Care in Africa

**DOI:** 10.1007/s11948-026-00616-w

**Published:** 2026-08-01

**Authors:** Kirubel Manyazewal Mussie

**Affiliations:** 1 Oxford Institute of Population Ageing, University of Oxford, Oxford, UK; 2 College of Health Sciences, Addis Ababa University, Addis Ababa, Ethiopia

**Keywords:** Artificial intelligence (AI), Clinical decision-making, Ethical challenges, The moral ecology of care, Relational impact assessment

## Abstract

Artificial intelligence (AI) is increasingly promoted as a tool to enhance clinical decision-making and thus improve the quality of healthcare. While much of the emerging scholarship on AI and healthcare in Africa has focused broadly on opportunities and systemic challenges, what remains underexplored is the specific application of AI to clinical decision-making. This paper contributes to addressing this gap by offering a conceptual and critical analysis of AI-based clinical decision support systems (AI-CDSS) in African contexts. Drawing on philosophical accounts of medical reasoning and relational moral frameworks such as Ubuntu, the paper draws on the moral ecology of care and shows that algorithmic systems can reconfigure epistemic authority, redistribute responsibility, and risk marginalising context-sensitive and relational dimensions of care. The paper further argues that AI systems are better understood as socio-technical mirrors that reflect and amplify existing human values, institutional arrangements, and power asymmetries. Moving beyond the algorithm, it proposes a shift toward relational and context-sensitive AI governance, including the development of relational impact assessments, the redistribution of responsibility across the AI lifecycle, and the co-production of knowledge with local stakeholders. While focusing on African clinical contexts, the analysis offers broader insights for global debates on AI ethics and clinical decision-making.

## Introduction

Artificial intelligence (AI) is becoming increasingly integrated into healthcare and shaping how clinical decisions are made, resources are allocated, and patient outcomes are achieved. Globally, algorithms are being deployed for diagnostics, predictive modelling, treatment optimisation, and workflow automation (Manyazewal et al., 2023; Olawade et al., 2024). Evidence suggests that AI can improve patient outcomes by analysing large datasets, detecting subtle clinical patterns, and providing timely, evidence-based recommendations to health practitioners (Gala et al., 2024). These developments are frequently framed as necessary responses to mounting pressures on healthcare systems, including rising costs, practitioner burnout, diagnostic error, and the exponential growth of biomedical data.

In many African health systems, the appeal of AI is particularly pronounced. Chronic shortages of health care professionals, uneven distribution of specialist expertise between urban and rural regions, fragmented data infrastructures, and a persistent dual burden of communicable and non-communicable diseases have created the need for more technological interventions (Alaran et al., 2025; Ndembi et al., 2025). AI-CDSS are often presented as tools to extend expertise, improve resource capacity, and support resource allocation. In this context, AI is frequently described as offering the possibility to overcome infrastructural limitations, which allows health systems to bypass certain developmental constraints through digital innovation.

However, these technological interventions are not without limitations. The excitement surrounding AI in healthcare often obscures deeper critical questions around, for example, ethics, equity, cultural fit, and governance. Notable drawbacks include loss of empathy, system failures, and concerns regarding trust, safety, and the quality of compassionate care (Chivilgina et al., 2021; Cordero, 2024; Li et al., 2025; Olawade et al., 2024). In CDSS – including softwares (automated or semi-automated) that guide or assist health care providers – the risks of using AI can be high: they affect diagnosis, treatment, and outcomes for vulnerable patients. This is particularly true in the African context, where health systems often contend with resource constraints, workforce shortages, and a high burden of both infectious and chronic diseases (Alaran et al., 2025; Manyazewal et al., 2024).

International guidelines, such as the World Health Organisation’s (WHO) framework on the ethics and governance of AI for health, emphasise principles of transparency, inclusiveness, human oversight, and responsibility (WHO, 2024). Similarly, a growing body of AI ethics scholarship has expanded beyond narrowly technical concerns to address questions of fairness, social justice, participation, solidarity, and power in the design and deployment of algorithmic systems (Corrêa et al., 2023; Jobin et al., 2019; Mittelstadt, 2019; WHO, 2021). While these developments represent important advances, comparatively less attention has been devoted to how AI-CDSS may reshape the relational and interpretive dimensions of clinical judgement itself, particularly within many African healthcare settings. This includes questions about how algorithmic systems influence the exercise of professional judgement, the distribution of epistemic authority, and the moral relationships through which clinical care is delivered. In contexts marked by resource constraints, plural moral commitments, and enduring global asymmetries in digital health innovation, these issues carry distinctive ethical significance (Birhane, 2020; Mussie et al., 2022; Sekalala & Chatikobo, 2024).

The title of this article, “Beyond the Algorithm”, signals that we should look past the seductive promise of technological optimisation to examine what is at stake when algorithms enter clinical decision procedures in African settings. This requires moving beyond the algorithm as a discrete tool and instead examining its role within what can be described as the moral ecology of care. Clinical decisions are not merely outputs of data processing; they are situated moral acts embedded in relationships of trust, professional responsibility, institutional constraint, and sociocultural expectation (Galasiński et al., 2023; Muyskens et al., 2025). When algorithmic systems enter this space, they go beyond information management and participate in defining which reasons are salient, how uncertainty is handled, whose judgement carries authority, and how responsibilities for outcomes are distributed. I suggest that AI systems introduce forms of rationality that prioritise quantification, standardisation, and optimisation in ways that may compress or even dismiss relational and context-sensitive dimensions of clinical judgement. And in under-resourced settings where reliance on algorithmic outputs can easily become the norm, AI can create situations where health practitioners become accountable for decisions grounded in algorithmic reasoning they did not fully control or author.

Existing internationally and widely used AI ethics frameworks should not be understood as indifferent to relational concerns. Contemporary guidelines invoke values such as participation, inclusion, solidarity, community engagement, social justice, and collective well-being as important components of responsible AI governance (Corrêa et al., 2023; Jobin et al., 2019; UNESCO, 2021; WHO, 2021). Similarly, recent advances on AI governance in African contexts have emphasised Ubuntu, participatory governance, and forms of accountability sensitive to local contexts (Gwagwa et al., 2022; Mahomed et al., 2026). Therefore, the concern advanced in this paper is not that relational considerations are entirely absent from existing frameworks. Rather, it is that they are often articulated as high-level ethical principles while receiving comparatively less attention as objects of evaluation in their own right. In clinical settings, the crucial question is not only whether governance frameworks endorse participation, solidarity, or inclusion, but also whether AI systems reshape the relationships through which trust, responsibility, judgement, and care co-exist in everyday practice. This concern is especially important in contexts where communal obligations, shared vulnerability, and relational understandings of personhood play an important role in clinical decision-making.

This paper makes a more specific contribution than the general claim that clinical decision-making is relational or that Ubuntu is relevant to AI ethics. Its distinctive argument is that AI-CDSS in many African healthcare settings create a form of relational displacement: they can preserve formal human oversight while shifting epistemic authority, responsibility, and the criteria of good clinical judgement away from locally embedded moral relationships. The paper develops this argument by bringing together three literatures that are often treated separately: philosophical accounts of clinical judgement, African relational ethics, and AI governance. Therefore, its contribution is not simply to “apply Ubuntu” to AI, but to show how AI-CDSS should be evaluated according to whether they preserve or erode the moral ecology of care. On this basis, the paper proposes a relational impact assessment as a complementary governance tool for evaluating how AI systems affect trust, interpretive agency, family and community involvement, responsibility, and local epistemic authority.

## What Counts as AI-Based Clinical Decision Support

This paper focuses primarily on AI-CDSS. These are tools designed to assist health care professionals in diagnostic, prognostic, triage, treatment, or resource allocation decisions by providing recommendations, predictions, alerts, or structured analysis (Bozyel et al., 2024; Sutton et al., 2020). Contemporary AI-CDSS increasingly rely on machine-learning techniques that identify patterns in large datasets and generate probabilistic outputs that may not be reducible to explicit rules programmed in advance (Adlung et al., 2021). Examples include diagnostic imaging systems, sepsis prediction models, mortality risk prediction tools, treatment recommendation systems, and, more recently, large multimodal models capable of integrating clinical, laboratory, and imaging data to support decision-making (WHO, 2024). At first glance, the ethical concerns discussed in this paper may appear not to be unique to AI. Health practitioners have long relied on guidelines, scoring systems, protocols, and other forms of decision support. Indeed, concerns about standardisation, professional autonomy, and the relationship between rules and judgements predate contemporary AI by several decades (Montgomery, 2006). Thus, it would be mistaken to suggest that AI introduces entirely unprecedented ethical challenges.

However, rejecting strong claims of AI exceptionalism does not mean that AI systems are ethically insignificant. Rather, AI-CDSS can intensify existing concerns and generate new governance challenges because of a particular combination of characteristics. First, many machine-learning systems operate through statistical associations that may be difficult for practitioners to independently interpret or scrutinise, thereby creating concerns regarding, for instance, opacity and explainability (London, 2019). Second, their performance depends heavily on the representativeness and quality of training data, which raises concerns when systems are developed using datasets that inadequately represent African populations and healthcare realities (Birhane, 2020; Li et al., 2025). Third, AI systems can be deployed at scale across institutions and clinical settings, which carries the risk of allowing particular assumptions about risk, diagnosis, or treatments to become embedded in healthcare infrastructures. Finally, AI-generated recommendations often require a degree of epistemic authority because they are perceived as data-driven, objective, or scientifically superior, even when their limitations remain poorly understood (Adlung et al., 2021; London, 2019).

Therefore, the concern of this paper is not that AI replaces human judgement altogether. Rather, it is that AI-CDSS can subtly alter the conditions under which judgement is exercised. They may preserve formal human oversight while simultaneously reshaping whose knowledge is regarded as authoritative, how responsibility is distributed, and what forms of reasoning become institutionally privileged. It is these relational and epistemic effects, rather than technological novelty alone, that motivate the analysis that follows.

## Clinical Decision-Making as a Moral Practice

Clinical practice is often perceived and presented as a technical and procedural activity whereby health practitioners, for example, gather evidence to diagnose patients, calculate risks associated with various treatment options, and select the intervention most likely to improve health outcomes. In this context, AI appears as a tool to advance evidence-based medicine by, among others, analysing vast datasets and generating probabilistic assessments that mostly exceed human cognition (Adlung et al., 2021; Sutton et al., 2020). The advancement referred to here resulting from the growing involvement of AI is in the conventional sense of advancing clinical practice through the growing role of digital health technologies. While this advancement is undoubtedly beneficial, the work of advancing the moral ecology of AI-CDSS warrants more attention. However, it is important to note that the claim that clinical judgement involves moral and relational dimensions is not itself new. Longstanding traditions within virtue ethics, care ethics, and medical ethics have argued that medicine requires moral discernment and responsiveness to the particular circumstances of patients rather than the mechanical application of technical rules (Montgomery, 2006; Noddings, 1984; Pellegrino & Thomasma, 1993). Therefore, the purpose of revisiting this literature here is not to establish that clinical decision-making is a moral practice, but to examine how the growing integration of AI into clinical decision-making may reshape the relational and epistemic conditions under which such judgement is exercised, particularly within many African healthcare settings.

The interpretive and moral character of clinical judgement, therefore, warrants more reflection. Clinical judgement is not simply a matter of applying rules or algorithms, as also argued by several notable experts in the field. For example, Eric Cassell argues that medicine concerns the suffering person rather than a set of abstract variables, which highlights that clinical judgement involves interpretive engagement with the lived experience of illness (Cassell, 2004). Similarly, Kathryn Montgomery argues that medical reasoning is not reducible to the mechanical application of rules, adding that “if clinical reasoning were simply a matter of pattern recognition or following an algorithm, a well-programmed computer might substitute for even the best physician” (Montgomery, 2006, p. 119). While what counts as the “best practitioner” is contested and extends beyond technical accuracy alone, for the purposes of this paper, it is not defined solely by diagnostic precision. It is rather defined by the capacity to deliver comprehensive and holistic care – care that integrates biomedical knowledge with interpretive sensitivity to the patient’s social, cultural, and relational context.

However, it is important to acknowledge that recent developments in AI complicate this picture. Machine-learning systems now demonstrate extraordinary capacities for pattern recognition, probabilistic prediction, and integration of heterogeneous datasets, sometimes outperforming human practitioners in narrowly defined diagnostic tasks (Adlung et al., 2021). At the same time, however, it must be noted that predictive performance does not equate to moral or relational understanding. Algorithmic systems operate through statistical abstraction and optimisation, not through experiential comprehension or a moral commitment to the patient (Coeckelbergh, 2020; London, 2019). Thus, on this account, excellence in clinical practice involves more than computational competence; it calls for moral sensitivity and attention to context, which are especially important when AI is involved in clinical decisions.

Clinical decisions are, therefore, embedded in relationships of trust and moral expectation (Morley & Floridi, 2020; Rubeis et al., 2018). Patients rely on practitioners not only for technical expertise but for judgement that is attentive to their particular needs, vulnerabilities, and even cultural backgrounds. Practitioners in turn often experience their role as extending beyond the strict delivery of biomedical interventions (Morley & Floridi, 2020). This is not merely a theoretical claim. In our qualitative work with frontline health care providers involved in drug-resistant tuberculosis (DR-TB) care in Ethiopia, for example, practitioners described “family-like” patient-provider relationships in which they empathised with patients and adapted treatment protocols to better fit their circumstances (Mussie et al., 2020). These relational and sociocultural understandings of clinical decisions are even more visible in (ethically) more sensitive areas such as old-age care. In our study of clinical decision-making in Ethiopia, both older patients and health professionals described decision processes strongly shaped by religious commitments, limited disclosure practices, and – even crucially – family involvement that could outweigh both patient preferences and practitioner recommendations (Mussie et al., 2024). Thus, clinical decision-making is not simply the output of data processing, but a moral practice negotiated within relationships of trust and sociocultural expectations.

The view that clinical decision-making is a moral practice is also supported by the retainment of human responsibility within decision-making processes, as the WHO asserts: “humans should remain in full control of health-care systems and medical decisions” (WHO, 2021, p. 25). Remaining in “full control”, however, should be understood as more than a formal requirement that the practitioner remain “in the loop”. It should preserve practitioners’ genuine epistemic agency: their capacity to participate meaningfully in the evaluation and application of clinical knowledge by interpreting, questioning, and, where necessary, overriding algorithmic outputs. Evidence on epistemic humility similarly argues that epistemic virtues in clinical practice are “formed through relationships” and “embedded in a social context such as a clinical encounter” (Muyskens et al., 2025, p. 1). Such agency involves more than the technical authority to accept or reject a recommendation; it requires the ability to assess its relevance and limitations in light of, for example, contextual knowledge, patient circumstances, and professional judgement (Montgomery, 2006; Muyskens et al., 2025). Epistemic agency is compromised when practitioners increasingly defer to algorithmic recommendations because of their perceived objectivity or institutional authority despite possessing reasons to question them. In such circumstances, human oversight may remain formally intact while becoming substantively weakened.

This concern is particularly significant in clinical contexts in Africa where AI systems are frequently adapted from datasets that inadequately represent local populations, disease profiles, and social realities, thereby increasing the risk that algorithmic recommendations reproduce or amplify existing health disparities (Li et al., 2025). Therefore, the integration of AI in such contexts is not merely a technical development but a potential reconfiguration of the normative foundations of clinical practice. Thus, I argue that recognising clinical judgement as a moral practice is essential if we are to assess not only whether AI systems function effectively, but also how they reshape care, responsibility, authority, and relationships in (many African) health systems. Moreover, drawing on all the above accounts, I want to emphasise that the significance of characterising clinical decision-making as a normative and interpretive practice for the present argument lies not in the claim itself but in what follows from it: if clinical judgement is partly constituted through relationships, interpretation, and moral responsibility, then AI systems cannot be evaluated solely in terms of predictive performance or technical effectiveness. Their ethical significance also depends on how they influence the conditions under which such judgement is exercised.

## The Limits of Algorithmic Care in Africa

### Conflict with Local Moral Values – The Case of Ubuntu

If clinical decision-making is understood as a moral practice, then any ethical analysis of AI-CDSS must pose the question: which moral frameworks can, and even should, illuminate the kinds of moral values at stake when decisions are mediated by algorithmic systems. The popular indigenous African value system called Ubuntu could be a typical example. In turning to Ubuntu, however, one must proceed carefully. African settings are not culturally homogeneous, and clinical practices vary across contexts. Accordingly, Ubuntu is not invoked here as an essentialist description of what Africans “really believe”, nor as a claim that Western contexts lack relational moral reasoning. Rather, Ubuntu is used as a philosophical resource – a well-developed family of relational moral theories, articulated and debated by African scholars, that helps foreground dimensions of moral agency and care that are often muted in globally dominant AI ethics discourse (Mbiti, 1969; Metz, 2017). In this sense, Ubuntu functions to clarify why some forms of “good care” cannot be fully captured by optimisation-oriented models of reasoning.

Many contemporary philosophical interpretations of Ubuntu treat personhood and moral agency as deeply shaped by relationships of mutual recognition, responsibility, and interdependence, although scholars disagree about precisely how much relationships contribute to personhood and moral development (Metz, 2017; Molefe, 2019). Therefore, the often-cited formulation – “a person is a person through other persons” – is best understood not as a slogan, but as a normative claim about the moral significance of human relationships. At the same time, Ubuntu should not be treated as a simple or uncontested solution to the ethical challenges raised by AI. Critics have argued that some interpretations risk romanticising community, obscuring conflict and power inequalities, or placing insufficient weight on individual rights and dissent (Marx, 2002; Matolino & Kwindingwi, 2013). Others caution against presenting Ubuntu as a singular African worldview given the moral, cultural, and political diversity that exists across African societies (Metz, 2017). For this reason, Ubuntu is used here not as a comprehensive description of African moral life but as a critical normative resource for examining how relationships shape moral agency, responsibility, and care.

Moreover, the presentation of Ubuntu in this article should not be interpreted as establishing a simple contrast between “African relationality” and “Western individualism”. Relational understandings of autonomy have long been developed within feminist ethics, care ethics, and mainstream bioethics, where persons are understood as socially embedded and shaped by relationships of dependence, care, and mutual recognition (Dove et al., 2017; Mackenzie & Stoljar, 2000). Thus, the relevance of Ubuntu lies not in establishing the existence of relational personhood – an idea already familiar in several bioethical traditions – but in placing strong emphasis on particular moral ideals of mutual recognition, solidarity, shared vulnerability, and communal responsibility as constructive features of ethical life (Metz, 2017; Molefe, 2019). From this perspective, the ethical question is not whether autonomy or relationality should prevail, but how clinical decision-making – including AI-mediated decision-making – can respect individual dignity while remaining responsive to the social relationships through which persons exercise agency and experience illness.

These debates are particularly relevant in clinical practice. For example, family and community involvement, where contextually relevant, may provide emotional support, facilitate caregiving, and strengthen trust, but they can also generate tensions, including pressure to conform to collective expectations, the marginalisation of patient preferences, or the silencing of vulnerable individuals whose interests diverge from those of family members or community authorities (Alfahmi, 2022; Dove et al., 2017; Sedig, 2016). Therefore, the relationality that Ubuntu facilitates should not be equated with moral consensus. Rather, the ethical significance of relationships depends on whether they promote recognition, respect, responsiveness, and meaningful participation while remaining compatible with protection against coercion and opportunities for dissent.

The relevance of these considerations for AI ethics is increasingly recognised in contemporary scholarship that interrogates the limits of supposedly “universal” AI ethics frameworks. Discourses on Ubuntu in global AI inclusion and governance debates argue that widely circulated ethical principles can carry implicit assumptions about the moral self-assumptions that may not adequately represent relational moral agency or collective responsibility (Gwagwa et al., 2022; van Norren, 2022; Yilma, 2025). This is important in clinical practice given that AI systems are not merely analytical tools but normative interventions that reshape how reasons are weighed and whose knowledge is treated as authoritative. Ubuntu thus provides a lens for assessing not only whether an AI tool is accurate, but whether it sustains the relational conditions of care. For example, an AI system might accurately identify a patient’s clinical risk and recommend an evidence-based intervention while remaining largely insensitive to the patient’s social circumstances, family obligations, religious commitments, or capacity to adhere to treatment. From a narrowly technical perspective, such a recommendation may be optimal. From a relational perspective informed by Ubuntu, however, the ethical adequacy of the decision would also depend on whether it supports relationships of understanding, responsiveness, and shared responsibility that enable care to be meaningfully practiced. Therefore, the concern is not only whether the recommendation is correct, but whether the process through which it is generated and applied remains attentive to the relational context in which illness and care are experienced.

### Moral Residue from the Lack of Relationality

Even when algorithmic recommendations are technically defensible, they may still leave behind moral residue – another risk that comes with algorithmic decision-making. Moral residue, which is commonly defined as a morally significant remainder that persists after making a justified decision in situations where important moral values conflict (ten Have & Patrão Neves, 2021), implies that the decision-making process has failed to address relational obligations, communal expectations, or context-specific vulnerability. This gap remains regardless of whether the decision-making process satisfies formal criteria such as efficiency or predicted benefit. This is particularly the case in settings where clinical decisions frequently involve trade-offs influenced by resource scarcity. For example, in many low-resource clinical environments, decisions about referral, diagnostic prioritisation, and treatment allocation cannot be separated from social realities such as transport barriers, caregiving duties, and similar contextual factors. These are precisely the sorts of contextual dimensions that algorithmic systems struggle to represent.

Algorithmic systems typically operate through abstraction. They translate illness into variables, assign weights derived from statistical correlations, and generate outputs that approximate optimal choices relative to a predefined objective function. Yet the ethical meaning of a clinical decision often lies not only in its predicted outcome but in the kind of relationship it expresses: whether it affirms dignity, sustains trust, recognises vulnerability, and is responsive to a patient’s social world. Approaching clinical judgement through a relational moral lens is thus critical to address this gap. A decision that is “optimal” in predictive terms may still be ethically incomplete if it disrupts relational obligations that are experienced as constitutive of good care, for example when family-based deliberation is treated as an obstacle rather than a meaningful moral practice, or when patient narratives are downgraded because they are not codable. Consider an AI-CDSS used to prioritise patients for referral from a rural clinic to a distant tertiary hospital. The system may classify a patient as lower priority because their biomedical risk score is moderate. But the practitioner may know that the patient is an older adult widow whose only caregiver is temporarily available, that travel requires family negotiation and financial sacrifice, and that delaying referral may make future access practically impossible. From the system’s perspective, postponement may be clinically “reasonable”. From the practitioner’s perspective, however, the decision leaves moral residue because it fails to register the relational and social conditions that make timely care possible. The ethical incompleteness lies not in the technical error but in the exclusion of morally relevant contexts.

This is one reason why the importation of AI ethics frameworks without deep contextual adaptation can be ethically problematic. Contemporary critiques argue that global AI ethics often relies on principles that sound universally appealing – such as fairness, transparency, accountability, etc. – but are not clear about how to resolve real moral decisions (Corrêa et al., 2023; Mittelstadt, 2019; Stahl, 2025; van Norren, 2022; Yilma, 2025). Fairness can mean equality, need, solidarity, or priority to the worst-off; transparency may serve health practitioners, patients, regulators, or vendors differently; accountability presumes institutional mechanisms that may not exist uniformly across health systems. A relational moral approach does not discard these principles, but it insists that their meaning is not purely technical. It is socially negotiated and morally shaped. Without explicit engagement with relational values, algorithmic systems risk compressing the ethical element into what can be optimised, while leaving relational obligations to be managed informally by health practitioners at the margins of the system.

### Algorithmic Authority and Epistemic Injustice

The authority that algorithmic systems have can be intimidating. Health practitioners who depend on AI tools for clinical decisions – regardless of the level or requirement of dependence – could gradually train themselves into viewing algorithmic suggestions as superior sources of clinical decisions. This is not to say that a “healthy” level of dependency is problematic per se, although this gives rise to questions such as what level is considered “healthy” and what context or who defines that. While providing further reflection on these issues sits outside the scope of this article, the characteristics and consequences of “unhealthy” dependency on algorithmic recommendations is necessary to reflect on. Health practitioners could pass the threshold of a “healthy” dependency when, for example, they overly depend on AI recommendations to the extent of doubting their professional suggestions even when those suggestions are more appropriate if they had been reviewed objectively or independently. Thus, algorithmic systems become superior not necessarily because they produce the best judgement, but simply because of the authority and recognition they have.

This superiority directly connects to questions of epistemic power. AI-CDSS do not merely provide information; they reorganise epistemic authority by shaping the social recognition of whose knowledge counts. Here, the concept of epistemic injustice is useful as coined by Miranda Fricker in her book “Epistemic Injustice: Power and the Ethics of Knowing” which describes the concept as “a wrong done to someone specifically in their capacity as a knower” (Fricker, 2007, p. 1). In AI-mediated clinical practice, epistemic injustice can arise when algorithmic outputs, often produced through models developed and validated outside African contexts, are treated as epistemically superior to local clinical judgement or patient testimony. This is not simply a matter of bias in data; it is also about authority. When a tool is labelled “evidence-based,” its recommendations can acquire normative force, which further shapes decisions and even constrains what counts as reasonable disagreement. Gradually, let alone disagreeing, practitioners may come to view even doubting as an intellectual luxury.

These dynamics create conditions for what can be described as ethical deskilling – which is not a loss of clinical competence, but a gradual narrowing of the space for health practitioners to exercise independent, context-sensitive moral judgement. While literature addressing AI in clinical practice in Africa rarely uses the exact term “ethical deskilling”, emerging works directly raise closely related concerns about diminished human agency, weakened clinical autonomy, over-reliance on AI recommendations, and accountability shifts in many African clinical contexts (Grancia, 2025; Naidoo et al., 2022; Ugar, 2026). Overreliance on automated outputs risks accepting erroneous recommendations despite contradictory evidence, especially when systems appear confident or authoritative. In overburdened clinical environments, the temptation towards overreliance can become structural rather than individual. Challenges such as time pressure, staffing shortages, and limited capacity for second opinions make reliance rational in practice even when it is ethically risky. Over time, this can diminish professional identity and shift the centre of clinical reasoning from interpretive deliberation to algorithmic authorisation, which further risks making the professional’s role procedural rather than substantive.

These concerns are intensified by the broader political economy of AI in many African health systems. Many AI tools are developed in the Global North, trained on datasets that may underrepresent African populations, and marketed as accessible solutions for “resource constraints.” Experts in African digital justice often describe these issues as forms of algorithmic or data colonialism, in which values and epistemic dominance travel through technological infrastructures (Birhane, 2020; Couldry & Mejias, 2019; Sekalala & Chatikobo, 2024). In clinical practice, this can translate into post-colonial technology flows where systems designed for different institutional realities are integrated into African contexts with limited local control over model governance, validation, or accountability. When adverse outcomes occur, responsibility often remains locally borne by practitioners and health institutions while authority to design AI systems, intellectual property, and economic gains remain external. This distribution raises justice questions not only about patient safety but, broadly speaking, about sovereignty, governance, and whose moral assumptions become integrated into clinical practice.

Consider, for example, an AI-CDSS used for different purposes such as to prioritise referrals, allocate scarce resources, or generate treatment recommendations in a resource constrained healthcare facility. The model may have been developed by an external technology company, trained predominantly on datasets collected outside of the local context, and validated based on performance criteria established elsewhere. Regardless, when a recommendation contributes to a poor clinical outcome, responsibility for explaining and managing its consequences typically falls upon local practitioners and healthcare institutions rather than those who designed the system. In such situations, health practitioners remain accountable for decisions that are shaped by technological infrastructures over which they exercise limited influence. Therefore, the concern is not simply that recommendations may be inaccurate, but that authority over important aspects of clinical reasoning can become embedded within externally developed systems while the responsibility for their consequences remains within local clinical relationships (Sekalala & Chatikobo, 2024; Sendak et al., 2020).

This asymmetry becomes particularly concerning and significant where local institutions have limited capacity to audit, modify, contest, or govern the models on which recommendations are based. Questions about acceptable risk thresholds, module performance, relevant outcome measures, and appropriate trade-offs may effectively be determined elsewhere while nevertheless shaping decisions made within many African healthcare settings. The resulting concern is not only one of patient safety but also of epistemic and political justice: who has authority to define what counts as good clinical reasoning, whose knowledge becomes embedded within technological systems, and whose moral assumptions are ultimately institutionalised through clinical practice.

The implication here is that meaningful governance of AI in African health systems cannot be reduced to safeguarding the technical aspects alone. It requires attention to relational moral agency, epistemic justice, and power. If indigenous moral values – such as Ubuntu – help illuminate why good care involves relational elements that cannot be fully optimised, epistemic injustice helps explain why AI systems can reshape authority structures in ways that marginalise local knowledge. Such tensions strengthen the central claim I make in this paper: moving “beyond the algorithm” requires recognising AI as a participant in moral and political ordering, not merely a computational aid. The normative task, then, is not simply to adopt AI responsibly, but to build conditions under which health practitioners and communities retain genuine agency over the values, priorities, and forms of care that AI systems institutionalise.

## The Ways Forward: Toward Relational and Context-Sensitive AI Governance

In the preceding sections, I have shown that AI-CDSS do not merely assist but actively reshape the moral and epistemic structure of care. Then the question that follows is not simply whether AI should be used or not. The reality is more complex. AI systems have clear potential to support clinical practice, particularly in settings where resources are limited and expertise is unevenly distributed. Thus, the challenge is not to eliminate these systems from African clinical environments, but to integrate and govern them in ways that do not displace the relational and moral dimensions of care. While providing a detailed strategy on how to address these challenges is beyond the scope of this article, I propose some measures based on my analysis.

However, I acknowledge that the concerns and recommendations outlined here are not exclusive to African clinical contexts. While this paper has focused on Africa (while also acknowledging the overwhelming diversity in the continent), many of the challenges discussed are likely to be present in other healthcare settings as well. What may differ is not necessarily the nature of these challenges, but the degree to which they are experienced and the structural conditions in which they emerge. In many African contexts, existing resource constraints, infrastructural limitations, and historical power asymmetries may intensify these issues and make their ethical implications more pronounced (Mussie et al., 2025; Sekalala & Chatikobo, 2024). Recognising this broader relevance does not diminish the importance of context-specific analysis. It rather brings perspectives from African clinical settings to global debates on AI and clinical decision-making.

### Rethinking “Intelligence”

One way forward, perhaps more ambitious than immediately actionable, is to reflect on how AI itself is understood within African healthcare contexts. Current conceptions of AI are not neutral technical descriptions; they are grounded in particular assumptions about intelligence, rationality, and decision-making – assumptions that prioritise abstraction, optimisation, and calculability. These models have undoubtedly enabled significant advances in clinical prediction and data analysis, yet they also carry implicit normative commitments about what counts as good reasoning and acceptable decision-making. Given that technological systems often embed the epistemic priorities and value systems of the contexts in which they are developed (Birhane, 2020; Couldry & Mejias, 2019; Gwagwa et al., 2022), when such systems are introduced into African healthcare settings, they do not arrive as neutral tools, but carrying particular ways of knowing and judging.

This raises a more fundamental question than is usually asked in debates on AI governance: not simply how AI can be adapted to local contexts, but whether its underlying conception of “intelligence” aligns with the relational, context-sensitive, and morally relevant ways of clinical judgement described in this paper. This is not to suggest that AI can or should be entirely redefined in isolation from its technical foundations. Rather, it is to argue that its integration into healthcare should involve a critical interrogation of the forms of reasoning it privileges and the kinds of knowledge it renders authoritative. In contexts where clinical practice is strongly linked with social relationships and communal obligations, treating intelligence as purely computational risks narrowing the scope of what counts as good care. Moving forward may, therefore, require not only contextualising AI systems, but also rethinking the normative assumptions they carry – shifting the focus from technical adaptation to ethical and conceptual alignment.

What would such alignment entail in practice? It does not necessarily require abandoning existing AI systems or developing entirely distinct forms of AI. Rather, it requires recognising that questions of model performance alone are insufficient for evaluating clinical decision support. AI systems should be designed, validated, and governed in ways that remain responsive to the social and moral contexts in which clinical decisions occur. This mainly includes preserving opportunities for practitioner interpretation and disagreement, incorporating locally relevant forms of knowledge, and evaluating how systems affect relationships of trust, responsibility, and care. Therefore, the argument is not that clinical judgement can never be computationally supported, but that some of its ethically significant dimensions (such as trust, practitioner interpretive agency, and relational autonomy, among others discussed throughout this paper) cannot be adequately assessed through technical performance measures alone.

### Assessing Relational Elements

It is important to rethink how AI systems are evaluated in clinical settings. Much of the current discourse focuses on performance metrics such as accuracy, efficiency, bias reduction, etc. These are undoubtedly important and have become central components of contemporary AI governance frameworks (Stahl et al., 2023; WHO, 2024). However, they do not fully capture what this paper has described as the moral ecology of care: the relationships, responsibilities, forms of judgement, and contextual sensitivities that give clinical decisions their ethical meaning. Similar concerns are raised by capability-sensitive approaches to health and well-being technologies, which argue that technological evaluation should attend to how technologies affect human capabilities, agency, and the conditions under which people are able to pursue valued forms of life (Jacobs, 2020). What is needed, therefore, is an additional layer of evaluation, one that explicitly examines how AI systems affect the relational conditions under which good care is delivered.

I propose that this could take the form of a relational impact assessment (RIA). A relational impact assessment should not be understood as a replacement for existing algorithmic, ethical, or human-rights impact assessments. Existing assessment frameworks play an important role in identifying risks related to issues such as safety, accountability, fairness, privacy, transparency, and legal compliance (Iunes Monteiro, 2025; Stahl et al., 2023). The argument advanced here is more specific. While existing frameworks primarily evaluate harms, benefits, rights, and governance arrangements, a relational impact assessment treats relationships themselves as an object of ethical evaluation. In clinical contexts, where trust, responsibility, interpretation, communication, and recognition are not merely desirable outcomes but constructive features of good care, these dimensions warrant explicit attention.

The central question of a relational impact assessment, therefore, is not simply whether an AI system functions effectively, but how it affects the relationships through which clinical judgement and care are practiced. Table 1 provides an illustrative sketch of what a relational impact assessment might examine. The broad domains are not intended as a definitive framework, but as examples of the kinds of relational and ethical dimensions that could be evaluated when assessing the impact of AI systems on clinical practice. The guiding questions identify the central normative concern within each domain, while the illustrative considerations indicate examples of issues that an assessment might examine when addressing those questions.

**Table 1:** Illustrative domains of a relational impact assessment (RIA)

Domain	Guiding question	Illustrative considerations
Trust	Does the system strengthen or weaken trust between patients and practitioners?	Patient understanding of AI involvement; ability of practitioners to explain AI-supported decisions; whether patients feel heard, respected, and treated as individuals
Epistemic agency	Can practitioners meaningfully interpret, question, and override recommendations?	Frequency of overrides; opportunities for disagreement; institutional expectations regarding AI use
Relational autonomy	Does the system support decision-making while respecting both individual preferences and social relationships?	Patient participation; family involvement; safeguards against coercion
Local knowledge	Are local expertise and contextual knowledge recognised as legitimate sources of knowledge?	Local validation; practitioner input; inclusion of patient narratives and contextual information
Responsibility	Are authority and accountability appropriately aligned?	Clarity of roles; accountability arrangements; documentation of decision responsibility
Equity and inclusion	Are certain populations disproportionately burdened or excluded?	Dataset representativeness; equitable access to AI-supported care; performance across demographic groups
Moral residue	Does the system leave unresolved relational or ethical burdens despite technically acceptable outcomes?	Practitioner moral distress; neglected social circumstances; unresolved care obligations

The purpose of a relational impact assessment is not to reduce relational values to a checklist or numerical score. Rather, it provides a structured process of ethical reflection that makes visible dimensions of care that are often overlooked when evaluation focuses primarily on technical performance or procedural compliance. In this respect, a relational impact assessment complements rather than competes with existing impact assessment frameworks. Moreover, and importantly, such an approach would also resist the tendency to treat ethical evaluation as a one-time pre-deployment exercise. The relational effects of AI systems are not static; they evolve as systems become integrated into everyday clinical practice. Relationships of trust, authority, responsibility, and dependency may change over time in ways that are difficult to anticipate during initial deployment. This suggests that evaluation should be iterative and reflexive rather than limited to a single assessment stage. Similar concerns have been raised in contemporary AI governance literature, where the absence of continuous oversight and adaptive governance mechanisms has been linked to the persistence of poorly adapted models that further widen inequalities and increase ethical and legal risks (Donia, 2025; Papagiannidis et al., 2025; Sendak et al., 2020). Likewise, the WHO emphasises that the ethical implications of AI systems evolve across contexts and over time, which require ongoing rather than one-off forms of assessment and governance (WHO, 2024).

This is particularly important in many African healthcare settings where relational dimensions are often not secondary features of clinical practice but central elements of how care is understood, negotiated, and experienced. If these dimensions are not explicitly considered in the evaluation of AI systems, there is a risk that they will gradually be displaced by forms of reasoning that privilege what is measurable, standardisable, and optimisable. Therefore, a relational impact assessment functions as a corrective lens that draws attention to moral and social consequences that might otherwise remain invisible. Whether this ultimately requires a distinct assessment framework or a substantial relational extension of existing impact assessments remains an open question. The central claim of this paper is more modest: relational effects deserve systematic evaluation within AI governance given that they shape the very conditions under which good clinical care becomes possible.

### Redistributing Responsibility and Authority

One of the more troubling dynamics I discussed in this paper is situations where practitioners could be held accountable for decisions that are significantly shaped by systems they did not design, do not fully understand, and cannot easily challenge. This creates a form of ethical asymmetry, where responsibility remains local while epistemic authority is external. Addressing this requires a clearer and more sensible alignment between authority and accountability, a concern also raised in Ubuntu-inspired analyses of responsibility gaps in AI-supported healthcare (Ferlito et al., 2024). If AI systems influence clinical outcomes, then responsibility must be distributed across the full chain of actors involved in their development and deployment, including developers, vendors, and institutions (Jobin et al., 2019). In African contexts, where many systems are imported and locally adapted rather than co-developed, the need for such alignment is particularly critical.

Closely related to this is the need to rethink the role of the human professional in facilitating clinical decisions in African clinical contexts. While some frameworks, including those of the WHO, emphasise keeping humans “in the loop,” this formulation can be misleading if interpreted too narrowly. Being formally involved in a decision-making process does not necessarily mean having genuine authority over it. A practitioner may technically retain the ability to override a recommendation, but in practice feel unable to do so due to various factors such as institutional expectations, perceived reliability of the system, or lack of (alternative) resources. This suggests that what is required is not merely human involvement, but human authority. Thus, efforts to ethically govern AI in Africa should deliberately include ways to preserve the practitioner’s capacity to interpret, question, and challenge algorithmic outputs.

However, preserving human authority should not be understood as sufficient in itself. Authority without adequate support can be equally problematic. If practitioners are to engage with AI systems critically, they must be equipped with the necessary forms of knowledge and competence to do so. This includes not only technical familiarity with how such systems function, but also cultural and contextual sensitivity that enables them to interpret algorithmic outputs in light of patients’ lived realities. Without such preparation, there is a risk that practitioners may either defer uncritically to algorithmic recommendations or exercise authority in ways that are poorly informed or misaligned with patients’ needs. Thus, human authority must be accompanied by continuous investment in clinically and culturally relevant training, reflexive practice, and institutional support structures that enable practitioners to navigate the complexities of AI-mediated clinical decisions with confidence and responsibility.

Beyond these adjustments, there is also a need to rethink how knowledge itself is produced and validated in the design and governance of AI systems that are used for clinical decision-making in Africa. Given that one of the risks associated with algorithmic authority is the marginalisation of local knowledge, there are questions about whose knowledge is considered legitimate and whose is rendered secondary. Addressing this requires moving beyond models of “local adaptation” of externally developed systems to approaches that prioritise the co-production of knowledge. This would require the participation of local practitioners, patients, caregivers, patient advocates, community representatives, health-system managers, technical developers, ethicists, legal experts, civil society organisations, and – where contextually relevant – religious leaders or traditional healers in the design, validation, and governance of AI systems in African clinical contexts (Birhane, 2020; Monteiro & Singh, 2025; UNESCO, 2021). Stakeholder inclusion should be determined by relevance to the care relationship, exposure to risk, position of local knowledge, capacity to represent affected groups, and safeguards against reinforcing domination or exclusion. Therefore, for example, religious bodies are not included because religion is universally authoritative across African contexts, but because in some settings religious commitments shape critical issues such as disclosure, family deliberation, end-of-life decisions, and trust in health institutions. In other settings, other stakeholders such as traditional healers, patient associations, disability groups, women’s organisations, or rural health workers may be more relevant.

This approach also, and more broadly, helps to address the bigger problem that I discussed previously: algorithmic or data colonialism. Indeed, this is with an acknowledgement that data colonialism is a far more complex and systemic challenge than can be fully addressed through the type of analysis proposed here. Nevertheless, efforts to advance the ethical use and governance of AI in African clinical contexts in general would benefit from investigating more questions such as who decides what outcomes are prioritised, who defines acceptable levels of risk, and who has the authority to question or reject a system. Without addressing these questions, AI risks becoming a vehicle through which external normative frameworks are integrated into local health systems without sufficient scrutiny.

### Rethinking AI Governance

Finally, governance should be understood not only as a technical or institutional matter, but also as a moral and political one. As I have discussed in previous sections, the integration of AI raises questions about, among others, what kind of care is valued, whose interests are prioritised, and how resources are distributed. These are not questions that can be addressed by technical optimisation alone. They require ongoing ethical reflection and, even importantly, deliberation inclusive of different varying opinions. In African contexts where health systems operate within complex social and historical conditions, such deliberation must involve not only policymakers and technical experts, but also practitioners and communities whose lives are directly affected by these systems.

At this point, it is also important to resist a framing in which AI itself is treated as the primary moral problem. I share the stand of experts who suggest that AI systems often function less as independent agents and more as mirrors of existing human practices, values, and limitations (Crawford, 2021; Green & Viljoen, 2020; Vallor, 2024). From this perspective, all the ethical challenges I discussed in previous sections are not created by AI in isolation, but reflect broader social, institutional, and epistemic conditions within which these systems are developed and deployed. If an AI system contributes to ethically problematic outcomes, responsibility ultimately lies with the human and institutional arrangements that design, procure, deploy, legitimise, and govern it (Coeckelbergh, 2020; Floridi & Sanders, 2004).

However, this does not imply that AI systems are ethically neutral or merely passive instruments. Once integrated into clinical practice, they can, among others, influence professional judgement, reshape patterns of attention, redistribute epistemic authority, and alter relationships of responsibility and care (London, 2019). Therefore, their ethical significance arises not because they possess moral agency, but because they participate in socio-technical systems that structure human action and decision-making. In this sense, AI systems do not merely reflect existing social and institutional conditions; they can also reinforce, amplify, and reconfigure them. Recognising this distinction helps avoid two equally problematic positions: attributing moral responsibility directly to AI systems on the one hand and treating them as ethically insignificant tools on the other. Therefore, governance requires attention both to the human choices incorporated into AI systems and to the ways these systems subsequently shape clinical practice.

Thus, the “moving forward” I envisage requires more than refining existing frameworks; it requires a shift in orientation. AI governance must move from a focus on controlling risks to engaging with the future of clinical decision-making as being increasingly shaped by AI. This involves recognising that AI systems are not neutral tools, but socio-technical actors that shape how care is understood, organised, and delivered in general. Ensuring that these systems support rather than undermine relational and context-sensitive care is, therefore, not simply a matter of better design, but of continuous ethical analysis and reflection. It is through such approaches that the integration of AI in African clinical practice contexts can move beyond the algorithm, toward forms of clinical practice that remain connected with, and even ground in, human judgement, relational responsibility, and moral agency.

## Conclusion

This paper has argued that understanding AI in clinical decision-making requires moving beyond questions of technical performance alone and examining how AI systems reshape relationships of care, epistemic authority, responsibility, and clinical judgement in clinical practice. Therefore, the integration of AI should be understood not simply as a matter of improving efficiency or predictive accuracy, but of reshaping the normative foundations of clinical decision-making itself. More specifically, the paper has argued that AI-CDSS may create a form of relational displacement in which formal human oversight is preserved while epistemic authority, responsibility, and the criteria of good clinical judgement are gradually reconfigured. By bringing together philosophical accounts of clinical judgement, African relational ethics, and AI governance, the paper proposes that AI systems should be evaluated not only according to technical performance but also according to their effects on the moral ecology of care. At the same time, the paper has argued that AI systems reflect and amplify existing human practices, values, and structural conditions. This perspective shifts the focus of ethical analysis from the technology alone to the broader socio-technical systems in which it is embedded. The challenges associated with AI in clinical decision-making are, therefore, as much about governance, power, and responsibility as they are about design and performance.

The proposals advanced in this paper – such as reconsidering the concept of intelligence, developing relational impact assessment tools, redistributing responsibility, and enabling the co-production of knowledge – are intended as starting points rather than final solutions. While focused on African contexts, they speak to wider global concerns about the future of clinical decision-making in an increasingly algorithmic world. Moving beyond the algorithm, then, is not a rejection of AI, but a commitment to ensuring that its integration remains responsive to human judgement, relational responsibility, moral agency, and the contextual realities within which care is delivered.

## Data Availability

Not applicable. This study did not generate or analyse datasets.
